# Supplementary material for: Antigen Cross-Presentation by Murine Proximal Tubular Epithelial Cells Induces Cytotoxic and Inflammatory CD8+ T Cells
Source: Cells. 2022 Apr 30;11(9):1510. doi: 10.3390/cells11091510 (PMC9104549; doi:10.3390/cells11091510)
Supplement: Supplementary file 1 [file cells-11-01510-s001.zip › cells-1696335-supplementary.pdf]

Supplemental material

Antigen cross-presentation by murine proximal tubular epithelial cells induces cytotoxic and inflammatory CD8$^+$ T cells

Alexandra Linke[1]*, Hakan Cicek[1]*, Anne Müller[1,2], Catherine Meyer-Schwesinger[3] Simon Melderis[2,4], Thorsten Wiech[5], Claudia Wegscheid[1], Julius Ridder[1], Oliver M. Steinmetz[2,4], Linda Diehl[1,2], Gisa Tiegs[1,2], Katrin Neumann[1,2]

[1]Institute of Experimental Immunology and Hepatology, University Medical Center Hamburg-Eppendorf, Hamburg, Germany

[2]Hamburg Center for Translational Immunology, University Medical Center Hamburg-Eppendorf, Hamburg, Germany

[3]Institute of Cellular and Integrative Physiology, University Medical Center Hamburg-Eppendorf, Hamburg, Germany

[4]III. Medical Clinic, University Medical Center Hamburg-Eppendorf, Hamburg, Germany

[5]Institute of Pathology, University Medical Center Hamburg-Eppendorf, Hamburg, Germany

*equally contributed

Table of content: 1 table, 7 figures

Table S1. Sequences of the primer used for analysis of mRNA expression.

Target	Forward primer<br>Reverse primer	Amplicon length	Annealing temperature
Actb	(fw) TATTGGCAACGAGCGGTTCC<br>(rv) GGCATAGAGGTCTTTACGGATGTC	180 bp	60°C
Mrc1	(fw) GGAGGCTGATTACGAGCAGT<br>(rv) TCCAGGTGAACCCCTCTGAA	87 bp	60°C
Sec61a1	(fw) TTCTGTGTCATCTTGCCGGA<br>(rv) TGCCAAACAGGGGGATCTGA	124 bp	60°C
Sec61b	(fw) TGAGTGCTCGGCAACTTCAC<br>(rv) GGGACAGGGCCCACTTTGA	282 bp	60°C
Sec61g	(fw) GCTCTCAATCCGCCATCCAA<br>(rv) AGGACTCAGCCACCCACAAT	237 bp	60°C
Vcp	(fw) TGACCCTCATGGATGGCCTA<br>(rv) TGTCAAAGCGACCAAATCGC	108 bp	60°C
Tap1	(fw) GGCTTACGTGGCTGAAGTCT<br>(rv) AATGAGACAAGGTTGCCGCT	123 bp	60°C
Tap2	(fw) TGTGCAGACGACTTCATAGGG<br>(rv) ATCTCCAGTTCTGTAGGGCCTG	199 bp	60°C
Lnpep	(fw) TTCGGCATGCTGTCATTCTT<br>(rv) GAGTTTTGTCTGTGACCTC ATTG	99 bp	60°C
Cybb	(fw) GCCAGTGTGTCGAAATCTG<br>(rv) AATTGTGTGGATGGCGGTGT	145 bp	60°C
Psmb8	(fw) GCCAAGGAGTGCAGGTTGTAT<br>(rv) GCCGAGTCCCATTGTCATCT	184 bp	60°C

SUPPLEMENTAL FIGURE S1

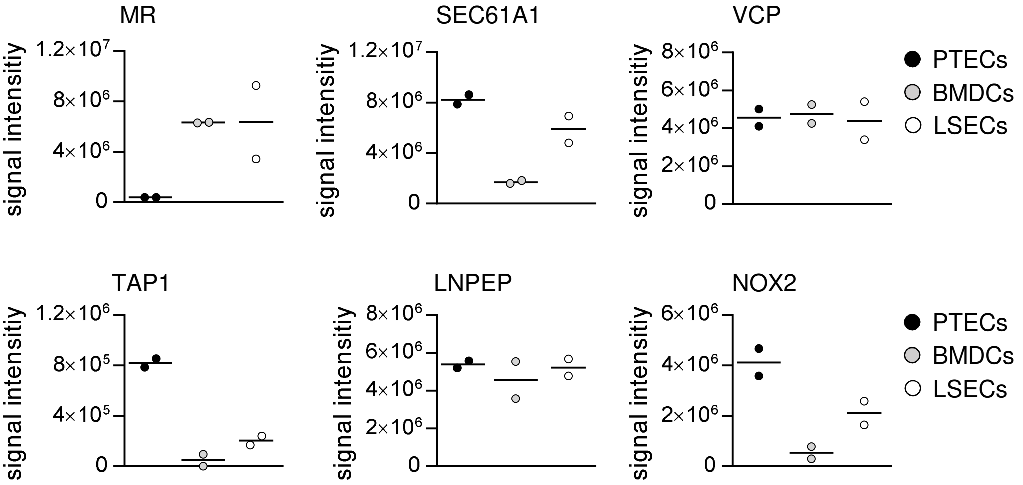

Supplemental Figure S1. Quantitative WB analysis. A densitometric analysis was done to otain realtive quantification of the analyzed proteins. Normalization was done in relation to the reference protein GAPDH. Medians show two experiments.

SUPPLEMENTAL FIGURE S2

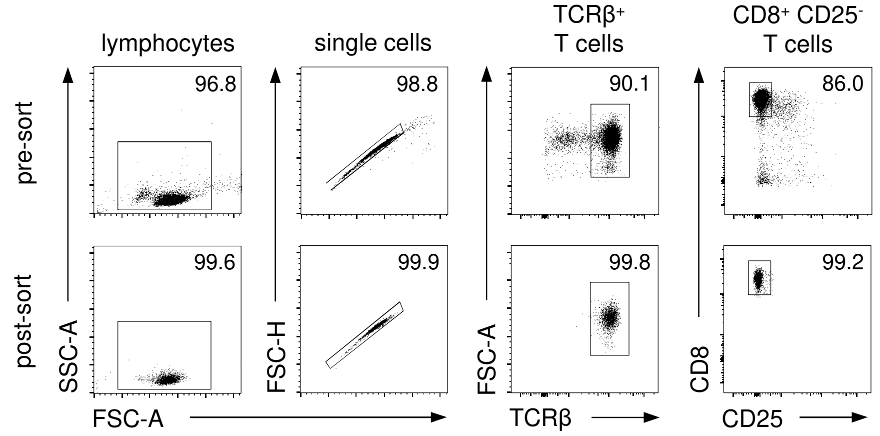

Supplemental Figure S2. Purity of isolated CD8⁺ T cells. CD8⁺ CD25⁻ T cells from spleen and lymph nodes of OT-I mice were enriched by MACS and purely isolated by FACS. Dot plots show representative data from at least 6 experiments.

SUPPLEMENTAL FIGURE S3

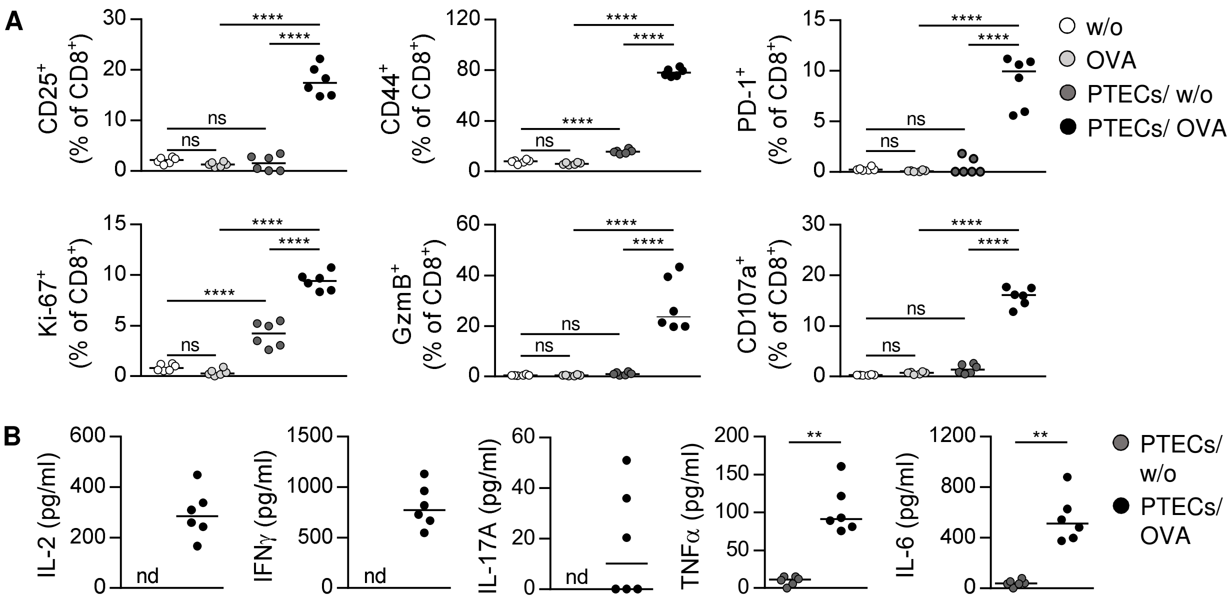

Supplemental Figure S3. Antigen-dependent activation of $CD8^+$ T cells by PTECs after 5 days of co-culture. OVA-specific $CD8^+$ $CD25^-$ T cells were cultured with PTECs or alone in presence or absence of OVA protein for 5 days. (A) $CD8^+$ $TCR\beta^+$ T cells were stained for CD25, CD44, PD-1, Ki-67, CD107a and GzmB, and analyzed by flow cytometry. (B) Cytokine levels were determined in culture supernatants. Medians are shown from two experiments. *p< 0.05; ***p< 0.001; ****p< 0.0001; ns: not significant; nd: not detectable; w/o: without OVA.

SUPPLEMENTAL FIGURE S4

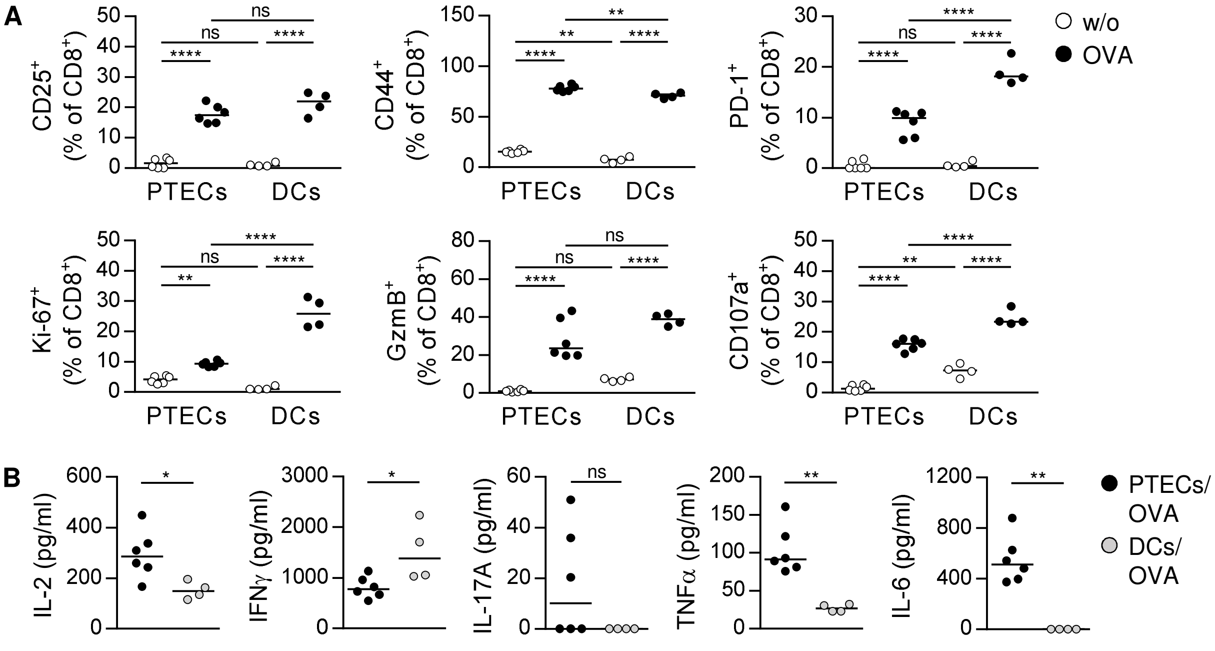

Supplemental Figure S4. Phenotype analysis of CD8+ T cells activated by PTECs or DCs after 5 days of co-culture. OVA-specific CD8+ CD25- T cells were co-cultured with PTECs or DCs in presence or absence of OVA protein for 5 days. (A) CD8+ T cells were stained for CD25, CD44, PD-1, Ki-67, CD107a and GzmB, and analyzed by flow cytometry. (B) Cytokine levels were determined in co-culture supernatants. Means are shown from 1-2 experiments. *p< 0.05; **p< 0.01; ****p< 0.0001; ns: not significant; w/o: without OVA.

SUPPLEMENTAL FIGURE S5

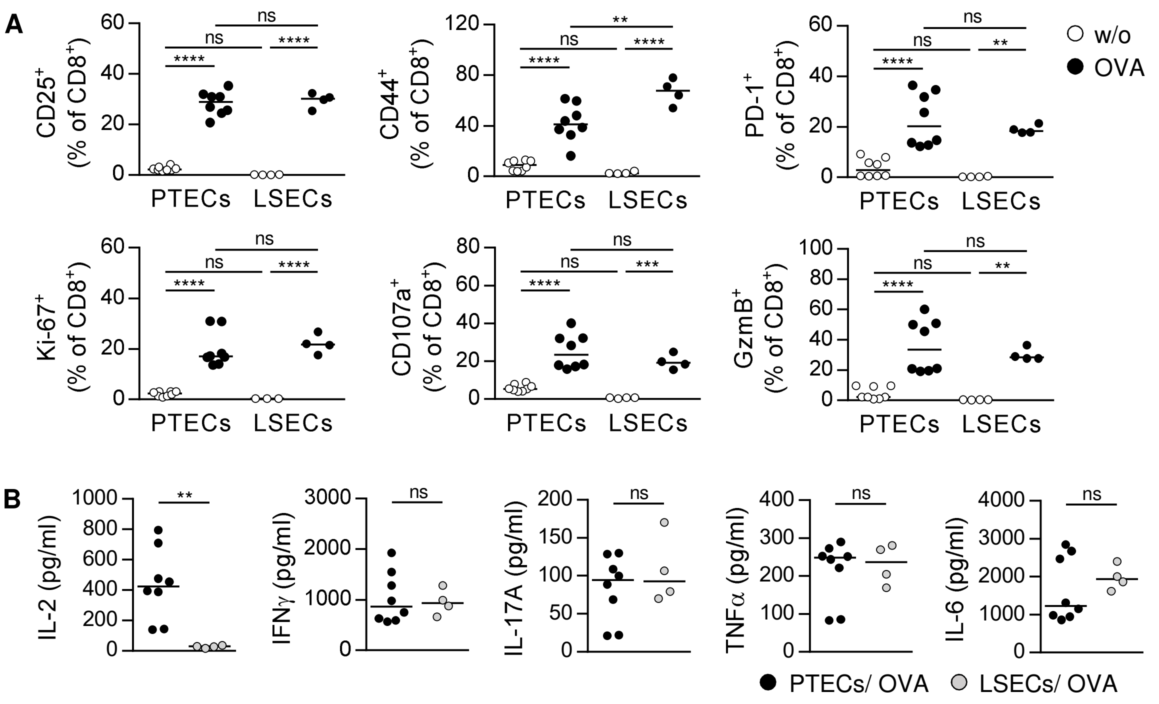

Supplemental Figure S5. Phenotype analysis of CD8+ T cells activated by PTECs or LSECs after 2.5 days of co-culture. OVA-specific CD8+ CD25- T cells were co-cultured with PTECs or LSECs in presence or absence of OVA protein for 2.5 days. (A) CD8+ T cells were stained for CD25, CD44, PD-1, Ki-67, CD107a and GzmB, and analyzed by flow cytometry. (B) Cytokine levels were determined in co-culture supernatants. Means are shown from 1-2 experiments. **p< 0.01; ***p< 0.001; ****p< 0.0001; ns: not significant; w/o: without OVA.

SUPPLEMENTAL FIGURE S6

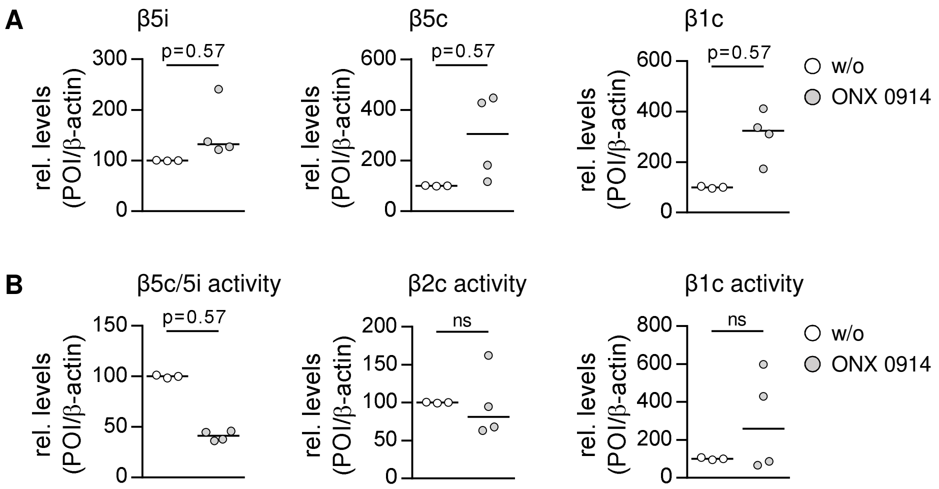

Supplemental Figure S6. Protein expression and catalytic activities of proteasome subunits. PTECs were treated with ONX 0914. (A) Protein expression of β5i, β5c and β1c was determined by WB analysis and depicted in relation to β-actin. (B) Activities of the different catalytic subunits were assessed by using ABPs. Medians of two experiments are shown. ns: not significant; w/o: without.

SUPPLEMENTAL FIGURE S7

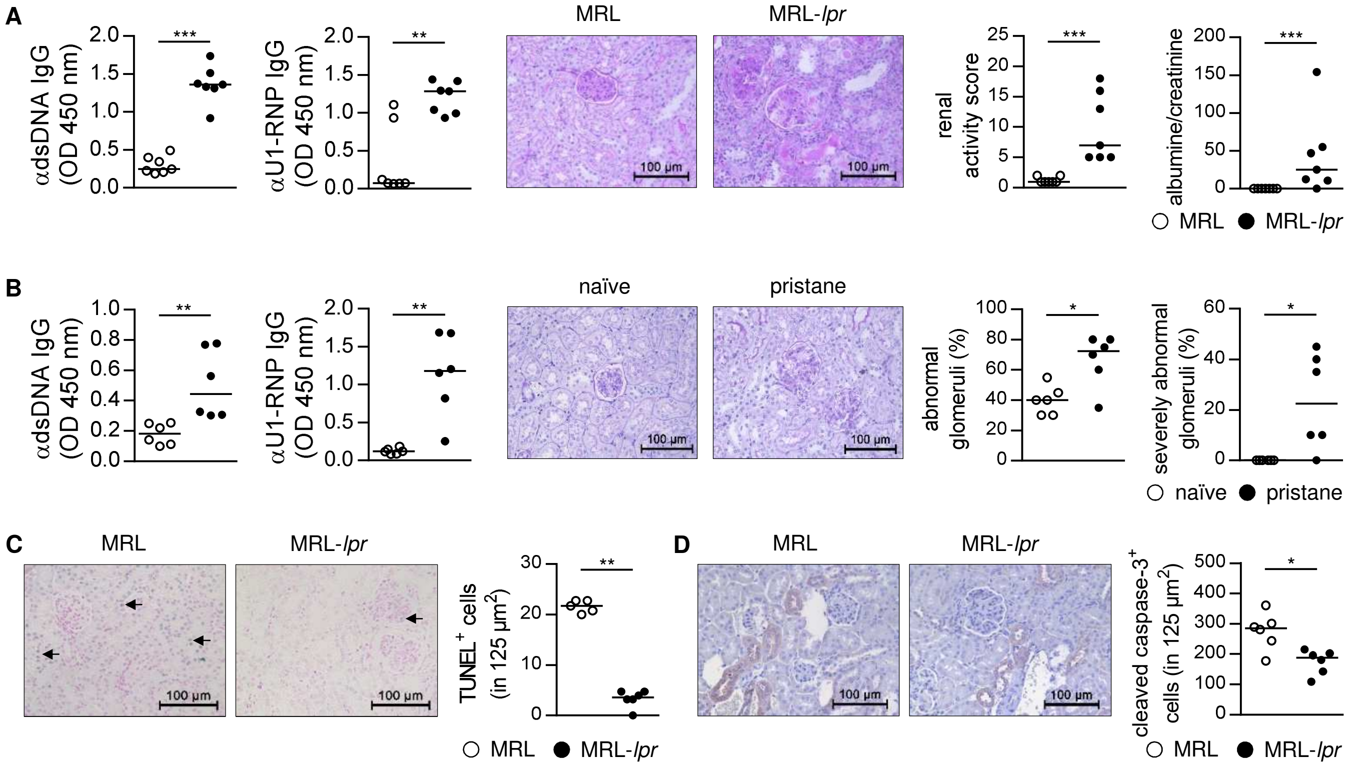

Supplemental Figure S7. Renal pathology in murine lupus nephritis models. (**A**) Lupus-prone MRL-*lpr* and MRL control mice were analyzed at an age of 15 weeks. Plasma autoantibody levels were determined by ELISA. PAS staining was performed in kidney sections. The activity score was assessed by a renal pathologist in a blinded manner. Albumin and creatinine levels were determined in urine. (**B**) WT mice were treated with pristane and analyzed 9 months later. Autoantibody levels were determined in plasma. PAS staining was performed in kidney sections. Frequencies of abnormal and severely abnormal glomeruli were assessed in a blinded manner. (**C**) Apoptotic cells were determined by TUNEL assay in kidney sections. (**D**) Numbers of cleaved caspase-3^{+} cells were assessed in kidney sections. Medians of 6-7 mice are shown. *$p < 0.05$; ***$p < 0.001$; αdsDNA: anti-double-stranded DNA; αU1-RNP: anti-U1-ribonucleoprotein.
